# Clathrin Adaptor Complex-interacting Protein Irc6 Functions through the Conserved C-Terminal Domain

**DOI:** 10.1038/s41598-019-40852-8

**Published:** 2019-03-14

**Authors:** Huajun Zhou, Giancarlo Costaguta, Gregory S. Payne

**Affiliations:** 1 Department of Biological Chemistry, David Geffen School of Medicine, University of California, Los Angeles, CA USA; 2 Gene Expression Laboratory, Salk Institute for Biological Studies, La Jolla, CA 92037 USA

## Abstract

Clathrin coats drive transport vesicle formation from the plasma membrane and in pathways between the trans-Golgi network (TGN) and endosomes. Clathrin adaptors play central roles orchestrating assembly of clathrin coats. The yeast clathrin adaptor-interacting protein Irc6 is an orthologue of human p34, which is mutated in the inherited skin disorder punctate palmoplantar keratoderma type I. Irc6 and p34 bind to clathrin adaptor complexes AP-1 and AP-2 and are members of a conserved family characterized by a two-domain architecture. Irc6 is required for AP-1-dependent transport between the TGN and endosomes in yeast. Here we present evidence that the C-terminal two amino acids of Irc6 are required for AP-1 binding and transport function. Additionally, like the C-terminal domain, the N-terminal domain when overexpressed partially restores AP-1-mediated transport in cells lacking full-length Irc6. These findings support a functional role for Irc6 binding to AP-1. Negative genetic interactions with *irc6∆* are enriched for genes related to membrane traffic and nuclear processes, consistent with diverse cellular roles for Irc6.

## Introduction

Clathrin-coated vesicles (ccv) mediate transport from the plasma membrane and between the *trans*-Golgi network (TGN) and endosomes. Clathrin forms the outer scaffold of the vesicle coat, linked to the membrane by adaptors. One major type of clathrin adaptors are the tetrameric adaptor complexes AP-2 and AP-1, which play roles in endocytosis and TGN-endosome transport, respectively. AP complexes consist of two large subunits, sometimes referred to as adaptins, and medium and small subunits. Through these subunits, AP complexes also serve to collect cargo and recruit other proteins that play a variety of roles in ccv formation.

In yeast, the adaptor complex-interacting protein Irc6 is a member of a conserved protein family defined by an N-terminal G protein-like domain and a C-terminal adaptin-binding domain^1,2^. The Irc6 orthologue in mammals, p34 (AAGAB), was initially discovered as a protein that binds AP-1 γ adaptin and AP-2 α adaptin subunits^3^. Haplo-insufficient mutations of p34 cause punctate palmoplantar keratoderma type 1 (PPKP1)^4–6^, an inherited skin disorder characterized by punctate thickening of the skin on the palms of hands and soles of feet. Electron microscopy of affected skin tissue revealed abnormal vesicle accumulation near the plasma membrane and a distended Golgi apparatus. Increases in epidermal growth factor receptor levels and phosphorylation were also observed, leading to a proposal that defective clathrin-mediated EGFR trafficking caused by p34 mutation contributes to the molecular etiology of the disease^4^.

Like p34, Irc6 interacts with AP-1 and AP-2^1,2^. Yeast cells lacking Irc6 (*irc6∆*) are defective in AP-1-dependent transport between the TGN and endosomes, but AP-2-mediated endocytosis appears normal^1,2^. TGN-endosome traffic can be restored in *irc6∆* cells by overexpressing p34, providing evidence for evolutionarily conserved function in this pathway^1^. Another interaction partner of Irc6, the Rab GTPase Ypt31, also acts in TGN-endosome transport. *In vitro*, Irc6 is required for Ypt31 to interact with AP-1, suggesting that Irc6 promotes ccv formation by bridging Ypt31 and AP-1^1^.

The Irc6p N-terminal domain exhibits a G-protein fold with highest similarity to small GTPases involved in protein trafficking^1,2^. However, unlike conventional GTPase domains, the N-terminal domain lacks both GTPase activity and high affinity binding to GTP^1^. Furthermore, mutations in conserved nucleotide binding motifs do not cause substantial defects *in vivo*. In contrast, a novel conserved BC-YY motif (beta-strand connecting YY motif), located in an accessible loop connecting beta strands in the N-terminal domain, contributes to Irc6 and p34 function in yeast.

The Irc6 C-terminal region is homologous to a domain family classified as “adaptin-binding” based on the ability of full-length p34 to interact with AP-2 α and AP-1 γ subunits. Subsequently, the Irc6 C-terminal domain was the first member of the adaptin-binding domain family to be tested for binding to AP-1 and AP-2, with inconclusive results. *In vitro*, the Irc6 C-terminal region bound both AP-1 and AP-2, as well as Ypt31^1^. However, in a separate study, a fragment containing the C-terminal region did not interact with the AP-1 γ subunit by 2-hybrid analysis^2^. In contrast, the N-terminal domain bound AP-1, AP-2, and Ypt31 *in vitro* and AP-1 γ in 2-hybrid assays^1,2^.

Consistent with observations that the C-terminal domain binds to the same spectrum of targets as full-length Irc6, over-expression of this domain alone from either Irc6 or p34 partially rescued the TGN-endosome transport defect caused by *irc6∆*^1^. The same test was not applied to the N-terminal domain because deletion of the C-terminal region from endogenous *IRC6* resulted in TGN-endosome defects that appeared to be more severe than deletion of the full gene.

Here, we extend characterization of Irc6 through domain-targeted mutagenesis, domain complementation assays, and bioinformatic analysis. Our results reveal that the last two residues of Irc6 are important for adaptor binding and TGN-endosome transport. We also report that the N-terminal domain can independently restore partial function in cells lacking full-length Irc6. Finally, analysis of a dataset of comprehensive pairwise genetic interactions links Irc6 function to both protein transport and nuclear processes.

## Results

### Mutations in the Irc6 C terminal domain reduce function

**Figure 1:**
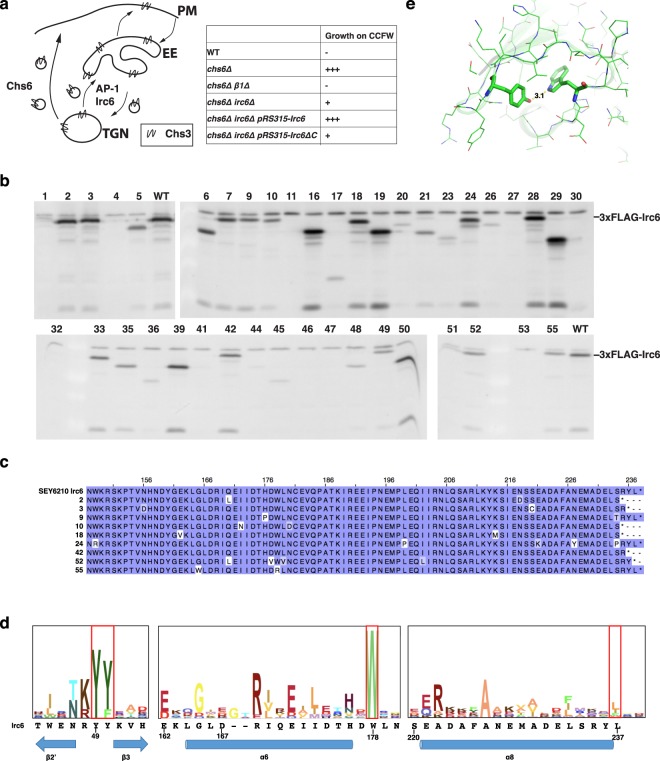
Identification of Irc6 C-terminal domain mutations. **(a)** Chs3p trafficking pathways and expected growth phenotypes of different strains on CCFW media. PM: plasma membrane; TGN: *trans*-Golgi network; EE: early endosome. Due to cisternal maturation of yeast Golgi compartments there may be little distinction between TGN and EE^36^. However, roles for AP-1 and Irc6 in forward and retrograde vesicular traffic between maturing late compartments of the Golgi would still apply. **(b)** Protein expression levels of Irc6 mutants. Extracts of cells expressing mutant or wild-type (WT) 3 × FLAG-Irc6p were analyzed by SDS-PAGE and immunoblotting with FLAG antibody. **(c)** Distribution of mutations in the Irc6 C-terminal region. Sequences of the indicated mutants are aligned with wild-type Irc6 and mutations are highlighted in boxes. Level of box shading is based on the BLOSUM62 substitution matrix; the lighter the shade, the less frequent the substitution. **(d)** HMM logo display^9^ of the BC-YY motif and two regions of the adaptin-binding domain, derived from 29 adaptin-binding family seeding proteins (Pfam10199). Irc6 sequences are aligned below the logo. Red boxes highlight the conserved di-tyrosine, tryptophan, and hydrophobic residue corresponding to the terminal Irc6 amino acid. Secondary structure is annotated at the bottom. α8 is predicted by PSIPRED. **(e)** Interaction between Y50 and W178. Region from the crystal structure of Irc6 highlighting the hydrogen bond between Y50 and W178 (PDB code: 3UC9). Distance in angstroms is shown.

To monitor Irc6 function in AP-1-dependent TGN-endosome transport, we used an assay for sensitivity to the chitin-binding dye, calcofluor white^1^ (CCFW; Fig. 1a). In wild-type cells, chitin in the cell wall, synthesized by chitin synthase Chs3, confers sensitivity to growth inhibition by CCFW^7^. In cells lacking Chs6, which is required to deliver Chs3 to the cell surface, Chs3 is retained intracellularly by AP-1-mediated cycling between the TGN and early endosomes. Intracellular sequestration of Chs3 in *chs6∆* cells reduces levels of cell wall chitin and confers resistance to growth inhibition by CCFW^8^. Perturbation of the AP-1 pathway in *chs6∆* cells, such as deletion of genes encoding AP-1 subunits or Irc6, releases Chs3 to the cell surface, restoring cell wall chitin and sensitivity to CCFW^1,2,8^. Thus, growth inhibition of *chs6∆* cells by CCFW can serve as a sensitive measure of defects in AP-1/Irc6-dependent localization of Chs3. Accordingly, to identify mutations in the Irc6 C-terminus that debilitate Irc6 function in the AP-1 pathway, we applied a plasmid-based targeted mutagenesis strategy and screened for mutants that conferred CCFW sensitivity in *chs6∆ irc6∆* cells (see Supplementary Fig. S1). For this approach, random mutations in the region of *IRC6* encoding the end of the N-terminal domain and adjacent full C-terminal domain were generated by error-prone PCR. Mutations were introduced by recombination into full-length *IRC6* under control of the native promoter on a low copy plasmid in *chs6∆ irc6∆* cells. The resulting transformants were then screened for CCFW sensitivity. Strains expressing plasmid-borne full-length wild-type Irc6 (aa1–237) and a C-terminal domain deletion mutant Irc6ΔC (aa1–179) served as positive and negative controls, respectively.

Out of 2.0 × 10^3^ transformant colonies, about 40 displayed reduced growth in the presence of CCFW compared to colonies of cells expressing wild-type Irc6. For each of these strains, the expression and size of Irc6 was determined by immunoblotting (Fig. 1b, Supplementary Fig. S2). Most mutants were compromised in expression and/or significantly truncated. For nine mutants that were expressed at relatively normal levels and size, the entire Irc6 coding sequence was determined. All but one mutant exhibited multiple changes (Fig. 1c). Whereas no missense changes were commonly repeated between mutants, six of nine lacked the terminal 1–3 amino acids. Notably, although most truncations were accompanied by missense mutations, mutant #42 (referred to hereafter as Y236*) was unaltered except for the absence of the terminal two residues. The high frequency of functionally defective mutants with changes that eliminate the most C-terminal residues suggests an important role for these amino acids in Irc6 function.

To guide interpretation of the missense mutations, we evaluated the relative conservation of residues in the adaptin-binding domain by aligning Irc6 to the proteins used in the seed alignment to build the adaptin-binding family (Pfam10199). In the graphical representations in Fig. 1d (and Supplementary Fig. S3), the height of each column of letters represents the deviation from the background frequencies of amino acids and serves as a measure of conservation of the column^9^. The height of each letter represents the frequency of that amino acid at that position. Among residues in the Irc6 adaptin-binding domain, tryptophan 178 (W178) is most highly conserved (Fig. 1d, Supplementary Fig. S3). Inspection of the Irc6 crystal structure (PDB code: 3UC9) reveals that W178 forms a hydrogen bond with Y50 (Fig. 1e), the second tyrosine in the BC-YY motif, which is also highly conserved (Fig. 1d, Supplementary Fig. S3). In mutant #55, which contains only two missense mutations, W178 is substituted by arginine. The other change in mutant #55, tryptophan for glycine at position 165, also affects a highly conserved residue (Fig. 1d).

**Figure 2:**
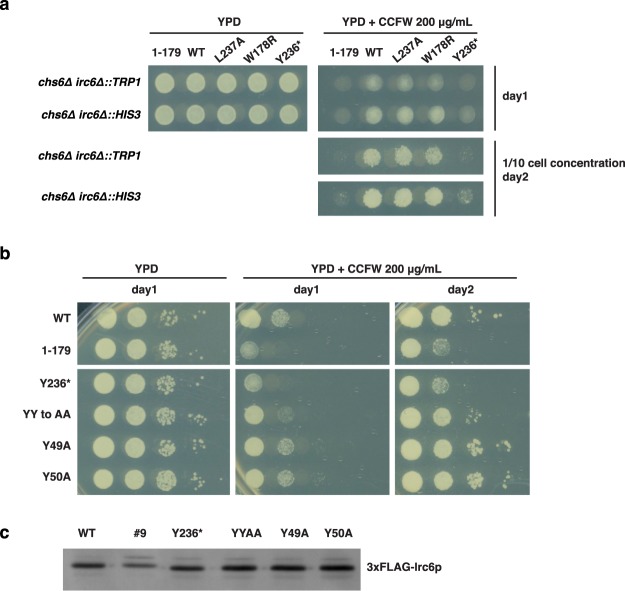
Irc6 C-terminal domain mutations that affect function. **(a)** Y236* is functionally defective. GPY4042 (*chs6∆ irc6∆::HIS3*) and GPY5086 (*chs6∆ irc6∆::TRP1*) cells expressing the indicated Irc6 mutants from a low copy plasmid were spotted onto media with or without CCFW and grown at 30 °C. Images are shown from the starting cell concentration after 1 day of growth (top panel) and from a 10-fold dilution of the starting cell concentration after 2 days of growth (bottom panels, images from a single plate). **(b)** Effect of Y236* is similar to deletion of the C-terminal domain. GPY4042 expressing the indicated mutants from a low copy plasmid were spotted as serial dilutions on media with or without CCFW and grown at 30 °C. Images were acquired after 1 and 2 days of growth. Panels in the same column are images acquired from a single plate. **(c)** Y236* and YY mutant proteins (YYAA, Y49A, Y50A) are expressed at normal levels. Protein expression levels were analyzed as described for Fig. 1b.

Since the W178R mutation affects a residue that interacts with the BC-YY motif, which is important for Irc6 function^1^, we assessed the effects of a single Irc6 W178R mutation using the CCFW assay. We also tested individual mutation of BC-YY motif tyrosines 49 or 50 to alanine. Additionally, an L237A single mutant was constructed to determine whether alteration of the terminal amino acid is sufficient to ablate Irc6 function. Although the position corresponding to L237 is not as highly conserved, there is a propensity for large hydrophobic residues (Fig. 1d, Supplementary Fig. S3). These mutants, and Y236*, which lacks the last two amino acids, were introduced on low copy plasmids into two independent *chs6Δ irc6Δ* strains and the cells were tested for growth in the presence of CCFW. Although Y236* was expressed at normal levels, mutant cells grew poorly, resembling *chs6∆* expressing *irc6∆C* (Fig. 2a–c, Supplementary Fig. S4). This result provides evidence that loss of the last two amino acids abolishes function of the Irc6 C-terminal domain. By comparison, cells expressing W178R or L237A Irc6 displayed robust growth in the presence of CCFW, similar to cells expressing wild-type Irc6 (Fig. 2a). The CCFW resistance of these strains indicates that W178R and L237A do not significantly impact Irc6 function. Similar results were obtained for Y49A and Y50A single mutants (Fig. 2b). In contrast, the YY-AA mutant conferred CCFW sensitivity that was intermediate between wild-type and irc6Y236* or *irc6∆C*. These findings suggest that the C-terminal domain plays a more critical role in Irc6 function than the BC-YY motif. Furthermore, the last two amino acids appear to be particularly important for C-terminal domain function, although it is unclear whether it is the length, the presence of both residues or just the penultimate residue that is required.

### The last two amino acids of Irc6p mediate interaction with AP-1 and AP-2

**Figure 3:**
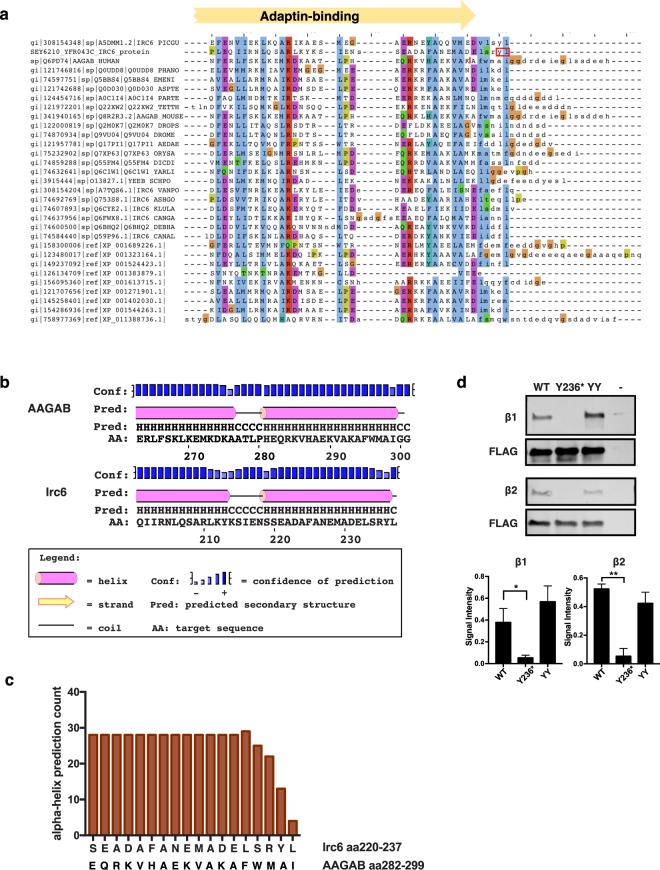
The last two amino acids of Irc6p are necessary for binding to AP-1 and AP-2. **(a)** Alignment of the C terminal region of Irc6p (row 2) with human p34 (AAGAB; row 3) and other adaptin-binding-domain family seeding proteins (derived from PSSM-ID: 313434). Consensus residues were highlighted using Clustal X color scheme. The yellow arrow indicates sequences that are part of the adaptin-binding domain as determined by the Conserved Domain Database. The full adaptin-binding domain extends to sequences upstream of those shown. The last two amino acids of Irc6 are outlined in red (row 2). The site in the AAGAB protein sequence affected by the C.870 + 1 G > A mutation is indicated by a vertical red line (row 3). **(b)** The Irc6 C-terminal domain is predicted to contain α-helices. PSIPRED secondary structure prediction of the Irc6 C terminal region aa201–237 and the corresponding region of p34. **(c)** Prediction of a C-terminal α-helix is conserved in Irc6 homologues. SPIDER2 secondary structure prediction of all 29 seed sequences of the adaptin-binding family (Pfam10119) aligned with the Irc6 and AAGAB C-terminal sequences. Shown are the number of sequences predicted to have α-helical properties at each sequence position. **(d)** Defect in interaction of Y236* and AP complexes. Flag-tagged wild-type (WT) and mutant versions of Irc6 expressed from a low copy plasmid in *chs6∆ irc6∆* cells (GPY4042) were immunoprecipitated from cell lysates, subjected to SDS-PAGE, and analyzed by immunoblotting with antibodies to β1 (AP-1), β2 (AP-2), and FLAG. GPY4042 carrying empty vector pRS315 was analyzed as a negative control (−). Quantification of the signals from three independent experiments are shown as bar plots. (*p value < 0.05; **p value < 0.01).

The final two amino acids of Irc6p are not included in the NCBI Conserved Domain Database (CDD)^10^ definition of the “adaptin-binding” domain, which ends at E232 in Irc6. However, alignment with adaptin-binding domain family members revealed significant conservation in the position corresponding to the Irc6 terminal leucine (Figs 1d and 3a, Supplementary Fig. S3). To gain insights into possible secondary structure in the C-terminal domain, we subjected the sequence to secondary structure prediction using PSIPRED^11^. The results predict that the penultimate tyrosine (Y236), but not the terminal leucine (L237), is part of an α-helix with the upstream 17 amino acids (Fig. 3b). PSIPRED analysis of the same region of p34 also predicts an α-helix, but includes the residue corresponding to Irc6 L237 in the helix. We also applied secondary structure prediction by the iterative deep learning neural network algorithm SPIDER2 to all seeding sequences in CDD used to model the adaptin-binding domain^12^. This yielded a moderately strong prediction of α-helix at the position of Irc6 Y236 that does not extend to L237 in the alignment (Fig. 3c). Thus, by these sequence and structure predictions, deletion of the last two residues in Y236* removes a conserved C-terminal leucine and a tyrosine that could be the terminal amino acid in an α-helix.

To determine whether the absence of the last two amino acids affects Irc6p binding to adaptor complexes, binding of mutant Y236* to AP-1 and AP-2 was assessed by co-immunoprecipitation. We also examined the effects of the YY-AA mutation on adaptor binding. Strikingly, AP-1 and AP-2 binding by Y236* was reduced to background levels (Fig. 3d). In contrast, the YY-AA mutation had minimal effects on binding to either adaptor complex. (Fig. 3d, Supplementary Fig. S5). The finding that truncation of the C-terminal two amino acids of Irc6 abolished interaction with AP-1 provides a mechanistic basis for the TGN-endosome transport defect in irc6Y236* cells.

### Irc6 N-terminal domain can function independently in TGN-endosome transport

**Figure 4:**
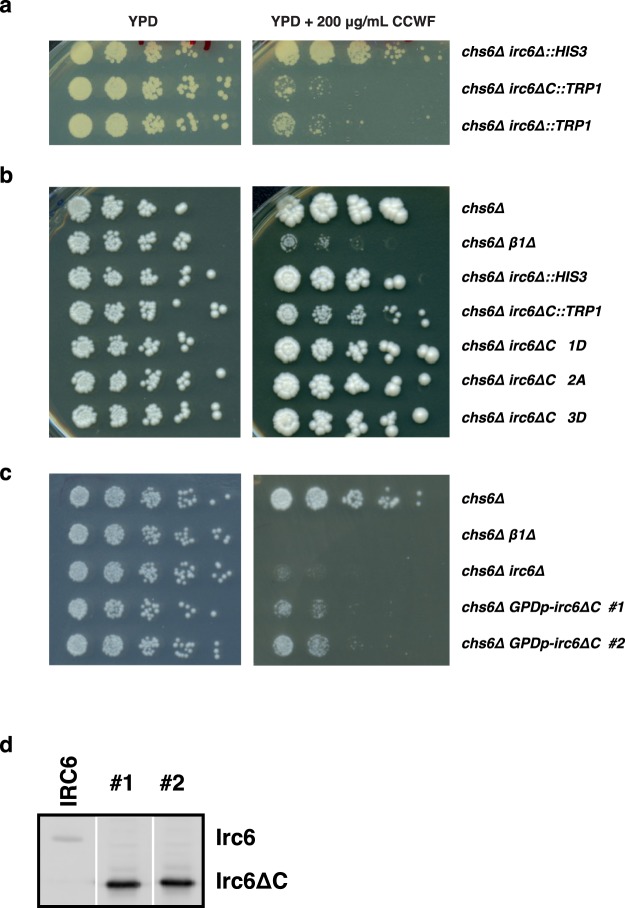
Functional analysis of the N-terminal G protein-like domain of Irc6. **(a**,**b)** Selectable marker effect on CCFW sensitivity. **(a)** Strains *chs6Δ irc6Δ::HIS3* (GPY4042), and *chs6Δ irc6ΔC::TRP1* (GPY4993), and *chs6Δ irc6Δ::TRP1* (GPY5086) were tested for growth in the absence or presence of CCFW as described in the legend to Fig. 2b. Images were acquired after 3 days of growth at 30 °C for CCWF. **(b)** The indicated strains were tested for sensitivity to CCFW as in **(a);** *chs6∆* (GPY3102), *chs6∆ β1∆* (GPY3103), *chs6Δ irc6Δ::HIS3* (GPY4042), *chs6Δ irc6ΔC::TRP1* (GPY4993), and *chs6Δ irc6ΔC* lacking a selectable marker (GPY5090-1D, 5091-2 A, 5092-3D). **(c)** Overexpression of Irc6 N-terminal domain partially substitutes for full-length Irc6. Indicated strains were tested for sensitivity to CCFW as in (a); *chs6∆* (GPY3102), *chs6∆ β1∆* (GPY3103), *chs6Δ irc6Δ::HIS3* (GPY4042). **(d)** Extracts from *IRC6* (*chs6∆*; GPY3102) and *chs6∆ GPDp-irc6∆*C strains (#1-2) were analyzed by immunoblotting with anti-Irc6 antibody. Lanes are from the same gel, rearranged in numerical sequence.

In our previous study of Irc6, *chs6∆* cells expressing Irc6 lacking the C-terminal domain from the native *IRC6* locus (*chs6∆ irc6∆C*) grew substantially less well in the presence of CCFW than *chs6∆* cells with a full deletion of *IRC6* (*chs6∆ irc6∆*)^1^. This observation led to the suggestion that the Irc6 N-terminal domain expressed by itself may act as an active inhibitor of AP-1-mediated transport. While generating additional strains to probe this possibility, we observed an effect of the *TRP1* selectable marker on CCFW sensitivity. The *chs6∆* strains in which *IRC6* was deleted and replaced with *TRP1* (*chs6∆ irc6∆::TRP1*) were substantially more sensitive to CCFW than *chs6∆* strains with *IRC6* replaced by *HIS3* (*chs6∆ irc6∆::HIS3*) (Fig. 4a rows 1 and 3, Supplementary Fig. S6, rows 1, 3–5). There was no difference in growth of these strains on media without CCFW. The basis for the selectable marker effects on CCFW sensitivity remains to be elucidated.

In the original report, the apparent inhibitory effect of the N-terminal domain was based on increased CCFW sensitivity of a *chs6∆ irc6∆C::TRP1* strain compared to a *chs6∆ irc6∆::HIS3* strain^1^ (Fig. 4a, rows 1–2; Supplementary Fig. S6). To address the possibility that the CCFW sensitivity difference could be due to distinct selectable markers, we compared strains with *irc6∆* or *irc6∆C* both marked with *TRP1*. With these alleles, *chs6∆ irc6∆C* and *chs6∆ irc6∆* strains exhibited equivalent growth on CCFW media (Fig. 4a rows 2–3; Supplementary Fig. S6). Furthermore, when *irc6∆C* was independently constructed without a selectable marker in *chs6∆* cells, these strains grew markedly better on CCFW than *chs6∆ irc6∆C::TRP1*, but similarly to *chs6∆ irc6∆::HIS3* (Fig. 4b, rows 3–7). In all cases, the mutant *irc6* alleles increased sensitivity to CCFW compared to wild-type *IRC6* (compare colony sizes in right-most dilutions, Fig. 4b, rows 1, 3, 5–7). These results indicate that expression of Irc6 lacking the C-terminal domain is deleterious to TGN-endosome traffic, but no more than the complete absence of Irc6.

We additionally tested the inhibitory effects of the N-terminal domain by constructing strains overexpressing Irc6∆C. If the Irc6 N-terminal domain actively inhibits AP-1-mediated traffic, then overexpression of Irc6∆C in *chs6∆* cells should lead to CCFW sensitivity that is even greater than that of *chs6∆ irc6∆* cells. Instead, Irc6∆C overexpression under control of a strong promoter improved growth on CCFW compared to cells carrying the full *irc6∆* deletion (Fig. 4c,d, Supplementary Fig. S7). Together with results from Fig. 2b, these findings provide evidence that, at normal expression levels, the C-terminal domain is critical for Irc6 function in TGN-endosome transport. However, overexpression of the N-terminal domain, like the C-terminal domain, can partially substitute for full-length Irc6.

### AP-1 localization in irc6∆ cells

**Figure 5:**
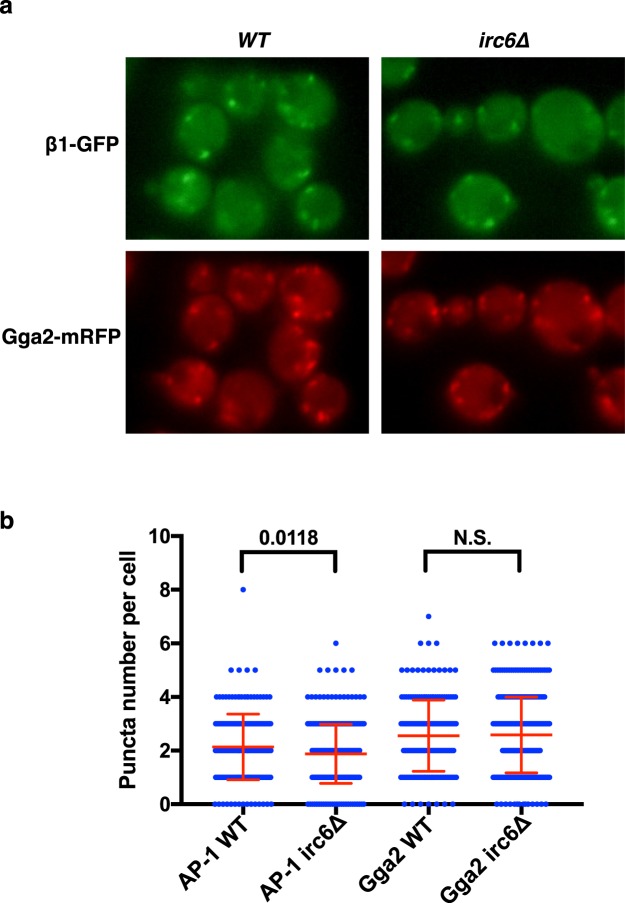
Adaptor localization effects of *irc6∆*. (**a**) Representative epifluorescence microscopy images of β1-GFP and Gga2-mRFP in *WT* (GPY3109) and *irc6Δ* (GPY4056). (**b**) Quantitative analysis of AP-1 (β1-GFP) and Gga2 (Gga2-mRFP) puncta in *irc6Δ* (GPY4056) versus wild-type cells (*WT*, GPY3109). Scatter plot of number of puncta per cell. Mean and standard deviation are shown in red. Means ± SEM and cell numbers (n): β1-GFP (AP-1) *WT* 2.14 ± 0.086, n = 203; AP-1 *irc6Δ* 1.88 ± 0.060, n = 331; Gga2-mRFP *WT* 2.56 ± 0.096, n = 190; Gga2-mRFP *irc6Δ* 2.58 ± 0.078, n = 332).

To determine whether loss of Irc6 affects AP-1 localization, we examined wild-type and *irc6∆* strains expressing AP-1 β1-GFP and the TGN clathrin adaptor Gga2-mRFP from the endogenous loci. Overall, mutant and wild-type strains exhibited a similar pattern of puncta, characteristic of TGN clathrin coats (Fig. 5a)^13,14^. There was a small but significant decrease in the number of AP-1 puncta per *irc6∆* cell compared to wild-type (*WT*: 2.14 ± 0.086, *irc6Δ*: 1.88 ± 0.060), whereas Gga2 puncta numbers were not affected (*WT*: 2.56 ± 0.096, *irc6Δ* 2.58 ± 0.078) (Fig. 5b). These results indicate subtle but specific effects of *irc6∆* on the frequency of AP-1 puncta.

### Ontology analysis of irc6∆ negative genetic interactions reveals enrichment in membrane trafficking and nuclear pathways

**Figure 6:**
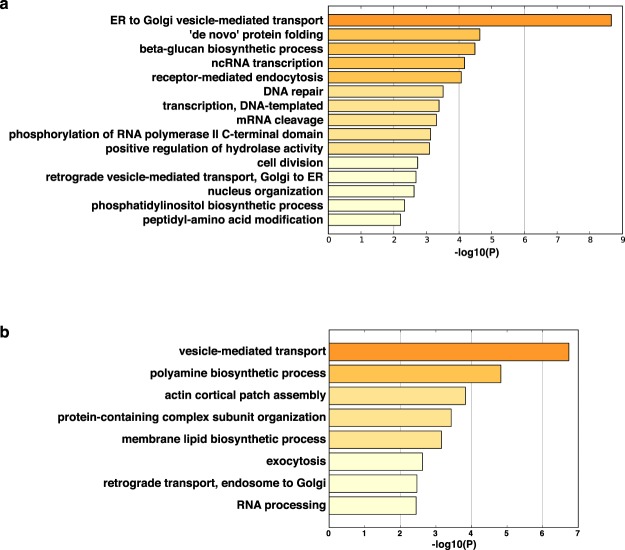
Gene Ontology analysis of genes that exhibit negative genetic interactions with *irc6∆* and *apm1∆*. Enriched Gene Ontology terms in the set of genes that are negative genetic interaction partners of *irc6∆* **(a)** and *apm1∆* **(b)** identified by Costanzo *et al*.^17^.

In addition to functioning in vesicle traffic, Irc6 has been implicated in genomic integrity based on an increase in DNA recombination centers in *irc6∆* cells and a decrease in telomerase-mediated healing of double strand DNA breaks in *irc6∆* cells^15,16^. As an unbiased approach to investigate the role of Irc6 in cellular processes, we took advantage of the negative genetic interaction dataset from a recent genome-wide pairwise analysis of mutations^17^. In this study, negative interaction between two mutations was defined as a more severe growth defect in the double mutant than expected from multiplying the growth defects of the single mutants. Negative genetic interactions tended to occur between genes in the same or related pathways, thereby providing an approach to identify potential functional connections^17^. Gene Ontology (GO) analysis of mutant genes that negatively interact with *irc6∆* revealed several processes related to vesicular transport. The most significantly enriched GO process is ER to Golgi vesicle-mediated transport (Fig. 6a), with 10 of the 48 negative interaction genes related to this pathway (see Supplementary Table S3). Also in the top five enriched processes are receptor-mediated endocytosis and beta-glucan biosynthetic process, which requires secretory pathway-mediated delivery of enzymes that synthesize beta-glucan components of the cell wall.

Notably, most of the other enriched GO terms from negative genetic interactions relate to nuclear functions, including DNA repair, a process that is active at DNA recombination centers that are increased in *irc6∆* cells (Fig. 6a). GO analysis of negative genetic interactions with a deletion of the AP-1 medium subunit, like *irc6∆*, yielded several processes related to vesicle transport (Fig. 6b). However, only one of the significantly enriched terms, RNA processing, was related to nuclear functions (Fig. 6b), suggesting that enrichment of genetic interactions of *irc6∆* with nuclear functions is not a general property of AP-1 pathway genes. Thus, the analysis from unbiased global genetic interaction maps supports functional roles for Irc6 in both membrane traffic and DNA recombination.

## Discussion

Irc6 and p34 are members of a protein family characterized by a bipartite organization with an N-terminal G-protein-like domain and a conserved C-terminal domain. Information on cellular roles played by this family has been limited to Irc6 and p34, which bind AP-1 and AP-2 and have been associated with clathrin-mediated membrane traffic in both yeast and humans^1–4^. Irc6 has also been implicated in DNA recombination and repair in yeast^15,16^. The studies reported here refine our understanding of Irc6 function by providing insights into the mechanism of AP binding, revealing functional redundancy between the two Irc6 domains, and describing connections of Irc6 to both membrane traffic and nuclear pathways in the genetic interaction landscape of yeast.

Conserved sequences in the C-terminal region of the p34/Irc6 family were designated as an adaptin-binding domain based on the properties of full-length p34, prior to any evidence that the C-terminal region of any family members interact with AP complexes. Subsequently, our group observed that both the C- and N-terminal domains of Irc6 interacted with AP-1 and AP-2 in affinity-binding assays^1^. However, another study reported that only the N-terminal domain bound to the γ adaptin subunit of AP-1^2^. Results reported here from our mutagenesis screen indicate that removing the Irc6 C-terminal two amino acids eliminates AP-1/AP-2 interaction and results in a Chs3 trafficking defect defined by CCFW sensitivity, comparable to a full deletion of *IRC6*. These findings provide additional support for a significant functional role of the Irc6 C-terminal domain in AP binding. The shared ability of overexpressed Irc6 and p34 C-terminal domains to partially suppress Chs3 sorting defects in *irc6∆* cells suggests that the AP-binding role is conserved^1^.

By sequence alignment and secondary structure predictions, the C-terminus of Irc6 ends with a conserved leucine (L237) preceded by a long α-helix that likely extends to the penultimate residue Y236. Bioinformatic analysis suggests a similar structure in the aligned sequences of other family members including p34. Whether the residues corresponding to Y236 and L237 play significant roles in the activities of p34 and other family members remains to be determined. It is worth noting that, consistent with this possibility, in cases of punctate palmoplantar keratodermas caused by p34 mutations, two Scottish families harbor a G to A mutation at the splice donor site following the codon for K290^4^. This mutation is expected to alter the C-terminal 22 amino acids of p34, disrupting the predicted α-helix in this region and removing the isoleucine corresponding to Irc6 L237.

The severe consequences on AP binding resulting from elimination of the terminal two residues were unexpected considering that the N-terminal domain also interacts with AP-1/AP-2. One possible explanation is that, in the native cellular context, the C-terminal domain plays a dominant role in AP binding. Alternatively, physical and functional engagement of AP complexes *in vivo* may require cooperative binding through both Irc6 domains. Both N- and C-terminal domains bind to AP-1, AP-2, and Ypt31 *in vitro*^1^. Furthermore, the results reported here indicate that the N-terminal domain, like the C-terminal domain, can partly substitute for full-length Irc6 when overexpressed. Based on these common physical and functional characteristics, we favor a cooperative binding model.

The studies reported here support the proposal that the BC-YY motif in the N-terminal domain is a common feature of the Irc6/p34 family and is required for function in Chs3 trafficking^1^. Our data indicate that the Irc6 YY mutant remains competent to bind AP-1 and AP-2. This finding suggests that the motif plays a distinct role in Irc6 function in the AP-1 pathway, perhaps contributing to interaction with another Irc6 binding partner such as Ypt31.

As an unbiased approach to identify pathways connected to Irc6 function, we analyzed enrichment of GO processes in a genome-wide dataset of pairwise negative genetic interactions. Three of the top five most significantly enriched GO processes associated with *irc6∆*-interacting genes involve membrane trafficking pathways: ER to Golgi vesicle-mediated transport, receptor-mediated endocytosis, and beta-glucan biosynthetic process. Enrichment of “ER to Golgi vesicle-mediated transport” likely reflects the deleterious impact of combining mutations that simultaneously affect transport at the *cis-* and *trans*-Golgi compartments. Similarly, negative consequences resulting from combining mutations that affect two distinct pathways through endosomes may underlie enrichment of the term “receptor-mediated endocytosis”. Considering the function of Irc6 in chitin synthase Chs3 trafficking, negative genetic interactions with genes associated with beta-glucan biosynthetic process can be attributed to cell wall integrity defects caused by coincident reductions in beta-glucan and chitin synthesis^18^.

In addition to membrane trafficking processes, prominent in the enriched GO terms of negative genetic interactions are nuclear processes - transcription and DNA repair - a finding that supports the connection of Irc6 to DNA recombination and repair. It is not yet clear whether nuclear defects in *irc6∆* cells are due to direct or indirect effects. However, it is intriguing that a C-terminal GFP-tagged version of p34 was distributed in both the nucleus and cytoplasm whereas an N-terminally tagged form was completely cytoplasmic^4^. Perhaps p34 (and Irc6) rapidly cycles into and out of the nucleus under normal conditions but the C-terminal tag interrupts the export step, shifting localization to the nucleus.

**Figure 7:**
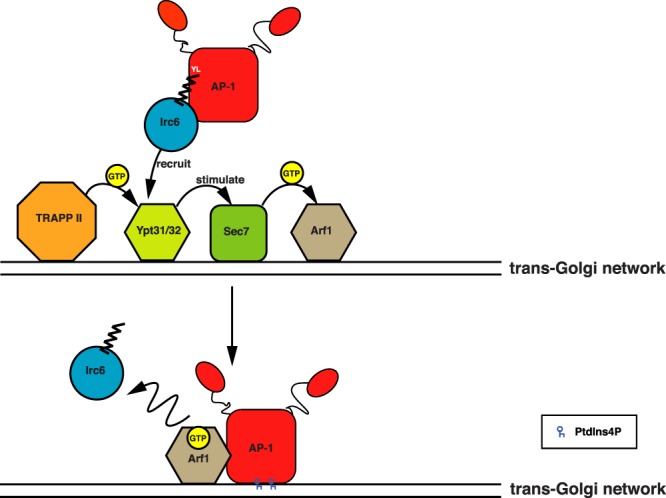
Model for Irc6 chaperone function in guiding AP-1 recruitment to the TGN. Irc6 N- and C-terminal domains (represented by circle and zigzag, respectively) cooperatively interact with the core domain of cytosolic AP-1. Irc6 binding to the GTP-bound form of Ypt31 positions AP-1 at the TGN. GTP-bound form of Ypt31 also stimulates GEF activity of Sec7, thereby activating Arf1. GTP-bound Arf1 and/or PtdIns4P anchor AP-1 to the TGN membrane and Irc6 is released.

Our analysis of Y236*, together with our prior studies, provide compelling evidence that AP-1 binding is a critical feature of Irc6 function in TGN-endosome traffic. Yet it remains unclear how this interaction, and the ability of Irc6 to link AP-1 to GTP-activated Ypt31, contribute to the mechanism of clathrin-mediated TGN-endosome trafficking. Several properties distinguish Irc6/p34 from many other clathrin accessory proteins, potentially offering clues to the mechanism of Irc6/p34 action. p34 is diffusely distributed in the cytoplasm, unlike the punctate localization characteristic of clathrin coat components including AP complexes^4^. In agreement with this localization, p34 interacts with cytoplasmic AP-1 and AP-2 and is not enriched in membrane fractions or clathrin-coated vesicles^4^. Also, in contrast to other AP-interacting accessory proteins, which bind to large subunit appendage domains that extend from the AP complex core, Irc6 and p34 interact with the AP core through the N-terminal regions of AP-1 γ and AP-2 α^2,3^. Based on these findings, it has been suggested that p34 acts as a chaperone for AP-1 and AP-2^3,4^. Extending this scenario to Irc6, we suggest that, as a chaperone, Irc6 helps guide localization of AP-1 to the TGN by binding activated Ypt31. AP-1 recruitment to the TGN depends on the GTP-activated form of Arf1^14^, however Arf1 is not likely by itself to target AP-1 to the TGN because it acts in vesicle formation throughout the Golgi complex. In these roles Arf1 is activated by different guanine nucleotide exchange factors at early (Gea1/2) and late (Sec7) compartments^19,20^. Importantly, Ypt31 was reported to be recruited to the TGN shortly after Sec7 and to stimulate Sec7 exchange activity on Arf1^20^. Thus, an Irc6-mediated interaction of AP-1 with Ypt31 could serve as the initial docking step of AP-1 with the TGN, so that AP-1 is positioned in close proximity to the TGN pool of activated Arf1 (Fig. 7). By this model, loss of Irc6 would reduce the efficiency of AP-1 targeting to the TGN, accounting for the decreased number of AP-1 puncta in *irc6∆* cells. Suboptimal recruitment by direct AP-1 interaction with Arf1 may explain the subtle effects of *irc6∆* on AP-1 localization and why the trafficking defects in *irc6∆* cells are not as severe as inactivation of AP-1^1^.

Phosphatidylinositol 4-phosphate (PtdIns4P), which is concentrated at the TGN, also contributes to AP-1 localization^13,21,22^. Considering that both Arf1 and PtdIns4P interact with the AP-1 core through the N-terminal region of the γ subunit^22,23^, it is tempting to speculate that once Irc6 delivers AP-1 to Ypt31, interactions of AP-1 with PtdIns4P and/or locally activated Arf1 displace Irc6 and anchor AP-1 to the membrane (Fig. 7). Release of Irc6 could also be facilitated by the conformational change to an open state that AP-1 undergoes upon Arf1/membrane binding^23^. In this scenario, Irc6 binding to cytoplasmic AP-1 might also help stabilize the closed state to prevent premature opening before recruitment to the membrane.

The model for Irc6 function in AP-1-mediated traffic proposed here represents a novel role for a regulatory factor in clathrin-coated vesicle formation, one that aligns with the distinctive properties of Irc6. The presence of the Ypt31/32 homologue Rab11 at the TGN in mammalian cells raises the possibility that a similar chaperone mechanism may apply to p34^24–26^. Whether this is the case, and whether elements of this paradigm extend to the function of Irc6/p34 in AP-2-mediated endocytosis, and even to potential roles in the nucleus, will await future investigation.

## Methods

### Yeast strains, media, growth assays

Yeast strains used in this study are listed in Supplementary Table S1. Strains were grown in YPD [1% Bacto yeast extract (Difco), Detroit, MI], 2% Bacto peptone (Difco), 2% dextrose plus additional 20 μg/mL alanine, 20 μg/mL uracil, and 224 μg/mL tryptophan] or SD media [0.67% yeast nitrogen base without amino acids (Difco), 2% dextrose] with the appropriate supplements. CCFW (Sigma-Aldrich, St. Louis, MO) was added to YPD agar plates at the concentrations indicated in the figures. For growth tests, cells were diluted to 1 × 10^6^ cells/ml and then serially diluted 5-fold (or 10-fold in Fig. 2) before dilutions were spotted onto appropriate agar plates.

Strains were derived from diploid yeast or haploid crosses using standard yeast genetic techniques. Deletions, promoters, and epitope and protein tags were introduced at the corresponding gene locus by homologous recombination^27,28^ and confirmed by PCR.

### Plasmids and yeast strain construction

Primers used in this study are listed in Supplementary Table S2. To generate the pRS315-3 × FLAG-Irc6 plasmid used for mutagenesis, the *IRC6* coding sequence with 500 bp flanking genomic sequences was amplified from GPY404.2 genomic DNA with primers containing SpeI and XmaI sites (HZ136, HZ137). 3 × FLAG tag was inserted into the N terminus of Irc6 by recombination PCR (HZ138, HZ139). The PCR product was then cut by SpeI and XmaI endonuclease and ligated into pRS315^29^. The resulting Irc6 containing plasmid contains ~300 bp promoter sequence, beginning at an endogenous genomic SpeI site. Error-prone PCR was carried out in the following mixture: 1x standard buffer (NEB), 0.2 mM each dATP and dGTP, 1 mM dCTP and dTTP, 2 μM Irc6 435 F primer (HZ140, positioned at codon for Lys152), 2 μM Irc6 814 R (HZ141, 100 bp downstream of stop codon) primer, 4.9 ng template DNA (Irc6 −300 bp to 500 bp), 0.5 mM MnCl_2_, 7 mM MgCl_2_, 5U Taq DNA polymerase (NEB) in total volume of 100 μL. DNA was amplified by incubations at 94°C × 1 min, 12 cycles of 94°C × 1 min, 54°C × 1 min, 72°C × 3 min. 20 μl product (~10 ng/μl) was co-transformed into 1.25 × 10^8^ GPY4042 cells with 100 ng pRS315-Irc6 pre-cleaved with BamH I (at aa168,169) and Nde I (at ~30 bp downstream of Irc6 stop codon). Separately, GPY4042 was transformed with pRS315-3FLAG-Irc6 and pRS315-Irc6 (1–179). Transformant colonies (≈2.0 × 10^3^) grown at 30 °C were replica plated to YPD + 200 μg/mL CCFW and YPD sequentially. After one day at 30 °C, colonies that grew poorly on YPD + CCFW but not YPD were retested for CCFW sensitivity. Plasmids from validated colonies were recovered and transformed into GPY4042, and the resulting transformants were tested for CCFW sensitivity. The full *IRC6* coding region in the recovered plasmids was sequenced using Irc6 814 R primer (HZ141).

To introduce the glyceraldehyde-3-phosphate dehydrogenase (GPD) promoter into the endogenous *irc6ΔC* locus, the GPD promoter and adjacent Kanamycin-resistance gene were amplified from pYM-N14 plasmid^27^ (HZ17, HZ18) and introduced into GPY4993. Haploid *chs6Δ irc6Δ::TRP1* strains were generated by *IRC6* replacement (HZ87, HZ88) in diploid GPY2288 and crosses of derived *irc6Δ::TRP1* haploids with GPY3102 or GPY4042. To construct *irc6ΔC* without an associated marker, a DNA fragment corresponding to *irc6ΔC* (aa 1–179) (HZ15, HZ16) was introduced with pRS315 [*LEU2*]^29^ into GPY4986 (*irc6Δ::URA3*), Leu + colonies were selected and screened for sensitivity to 5-fluororotic acid to identify Ura- transformants. *The irc6∆*C transformants were then crossed to GPY4042 to generate *chs6Δ irc6ΔC* strains.

### Protein expression immunoblotting and co-immunoprecipitation

For immunoblotting, cells carrying pRS315–3 × FLAG-Irc6 constructs were grown to exponential phase at 30 °C in SD media then 1 × 10^7^ cells were harvested by centrifugation and lysed by addition of 50 μl 2% SDS and incubation at 100 °C for 5 minutes. Samples were cleared by centrifugation at 20,000 × *g* for 20 minutes, subjected to SDS-polyacrylamide gel electrophoresis and immunoblotting. For co-immunoprecipitation, cells carrying pRS315-3 × FLAG-Irc6 WT/mutants were inoculated from SD media into YPD and grown to exponential phase at 30 °C. 5 × 10^8^ cells were harvested by centrifugation and converted to spheroplasts. Spheroplasts were resuspended in 200 μL binding buffer (20 mM HEPES, pH 7.0, 125 mM KAc, 0.4 M Sorbitol, 0.1% Triton X-100, 2 mM MgCl_2_, 0.5 mM EGTA, Proteinase Inhibitor Cocktail (Sigma-Aldrich), 0.5 mM PMSF, 0.5 mM DTT) and lysed by agitation with glass beads. Final volume was brought to 500 μL in binding buffer and then samples were subjected to centrifugation at 16,000 × *g* for 30 minutes at 4 °C. The resulting supernatant was incubated with 20 μL packed IgG‐Agarose beads (Sigma‐Aldrich) for 30 minutes at 4 °C followed by centrifugation at 16,000 × *g* for 15 minutes. The supernatant was then added to 20 μL packed ANTI-FLAG M2 magnetic beads (Sigma-Aldrich) for 2 hours at 4 °C. Beads were washed four times with binding buffer, once with wash buffer (20 mM HEPES pH 7.0, 0.1% Triton X-100, 0.5 mM PMSF, 125 mM NaCl), then bound proteins were eluted with three consecutive 5 min incubations with 20 μL wash buffer containing 250 µg/mL 3 × FLAG peptide. Eluates were pooled, mixed with 20 μL 5x sample buffer (50% glycerol, 0.25 M Tris-HCl pH 6.8, 10% SDS, 10% β‐mercaptoethanol, 0.03% bromophenol blue) and heated at 100 °C for 4 minutes, and analyzed by SDS-PAGE and immunoblotting. Antibodies used: FLAG (Sigma F3165). Irc6^1^, AP-1 subunit β1^30^, AP-2 subunit β2^30^. Quantification of the blot signals was carried out using Image Studio Lite 5.2. β1 and β2 signals were divided by corresponding FLAG signals, and then each was normalized as a fraction of the total sum of the ratios for each adaptor.

### Ontology analysis

Negative genetic interactions from Costanzo *et al*.^17^ were downloaded from https://thebiogrid.org/195888/publication/a-global-genetic-interaction-network-maps-a-wiring-diagram-of-cellular-function.html. The list of genes that exhibit negative genetic interactions with *irc6∆* was selected manually and submitted to metascape.org^31^, with all genes of *Saccharomyces cerevisiae* in the database as background.

### Sequence alignment and conservation analysis

The Irc6 sequence from SEY6210 was submitted to NCBI conserved domain (CD) search against CDD v3.16 using default settings^10^. The resulting alignment was downloaded, composed of the Irc6 query sequence and protein sequences that were used to build the Position Specific Scoring Matrix Model (PSSM-ID: 313434). Human p34 sequence (NP_078942) was added and manually aligned with mouse p34 protein sequence in Fig. 3a. The alignment was visualized by Jalview software^32^, using the default Clustal X colour scheme. The graphical representation of amino-acid conservation in Fig. 1d was achieved by submitting the alignment of the 29 seed protein sequences of adaptin-binding domain family (Pfam 10199) to skylign.org^9^. To achieve alignment of the N terminal region of the 29 sequences, clustal Ω^33^ was iteratively used to align the sequences. The height of each column is the information content, and the height of each letter is the relative frequency compared to other letters in the column. Only above-background residues are shown^9^. In Supplementary Fig. S3, 1145 adaptin-binding domain containing sequences, 167 P-loop NTPase and adaptin-binding sequences, and 161 Ras and adaptin-binding sequences were retrieved from NCBI protein database using the NCBI conserved domain architecture retrieval tool, were aligned by clustal Ω and visualized by skylign.org.

### Microscopy and image analysis

Cells were grown and prepared for microscopy as described in Fernandez *et al*.^14^. Images were captured using a 100 × α-Plan Fluar objective on a Zeiss Axiovert 200 M microscope (Zeiss, Hallbegmoos, Germany) with a Hamamatsu ORCA-ER camera (Hamamatsu, Hamamatsu City, Japan). Cell number and puncta number and intensity were quantified using the CellProfiler 3.0^34^ “speckle counting” pipeline, with an extra step in which background median intensity was subtracted prior to puncta intensity determination. Cell profiles from the CellProfiler pipeline were manually inspected and the occasional cases of misidentification of two cells as one cell were corrected. Data from two independent experiments were pooled, and p values were calculated by t-test using GraphPad Prism version 7.0a (GraphPad Software, La Jolla California USA, www.graphpad.com).

### C terminal domain secondary structure prediction

SEY6210 Irc6 sequence was submitted to the PSIPRED server (http://bioinf.cs.ucl.ac.uk/psipred/)^11,35^, and sequences that were used to build the CD domain model (PSSM-ID: 313434) were submitted to SPIDER2 server (http://sparks-lab.org/yueyang/server/SPIDER2/index.php)^12^.

## Supplementary information

Supplementary Information

## Data Availability

All data generated or analyzed during this study are included in this article (and the Supplementary Information files). Strains and plasmids are available upon reasonable request.
