# Supplementary material for: Clathrin Adaptor Complex-interacting Protein Irc6 Functions through the Conserved C-Terminal Domain
Source: Sci Rep. 2019 Mar 14;9:4436. doi: 10.1038/s41598-019-40852-8 (PMC6418106; doi:10.1038/s41598-019-40852-8)
Supplement: Supplementary file 1 — Supplementary Information [file 41598_2019_40852_MOESM1_ESM.pdf]

Supplementary Information

Clathrin Adaptor Complex-interacting Protein Irc6 Functions through the Conserved C-Terminal Domain

Huajun Zhou[1], Giancarlo Costaguta[2], and Gregory S. Payne[1]*

[1]Department of Biological Chemistry, David Geffen School of Medicine, University of California, Los Angeles

[2]Gene Expression Laboratory, Salk Institute for Biological Studies, La Jolla, CA 92037

*Corresponding Author: Department of Biological Chemistry, David Geffen School of Medicine, University of California Los Angeles, Los Angeles, CA, 90095, U.S.A. Telephone: 310-206-3121. Email: gpayne@mednet.ucla.edu

Figure S1

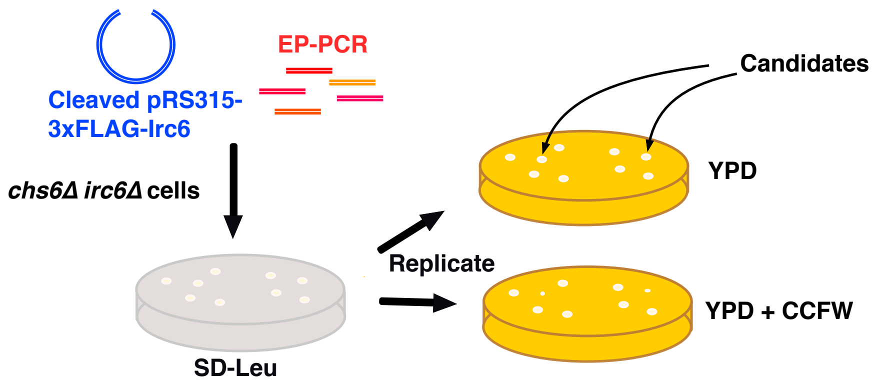

Figure S1. Screening strategy to identify mutations that affect the functionality of the Irc6 C terminal region. Error-prone PCR (EP-PCR) was used to generate mutant IRC6 fragments which were used to generate full-length Irc6 by transformation with gapped pRS315-3xFLAG-Irc6 into *chs6Δ irc6Δ* cells. Cells carrying recombinant plasmid were selected on SD -LEU media and screened for Irc6 function by replica plating onto YPD media with CCFW and YPD. Candidate mutants were identified as those colonies that were smaller on YPD + CCFW compared to YPD.

Figure S2

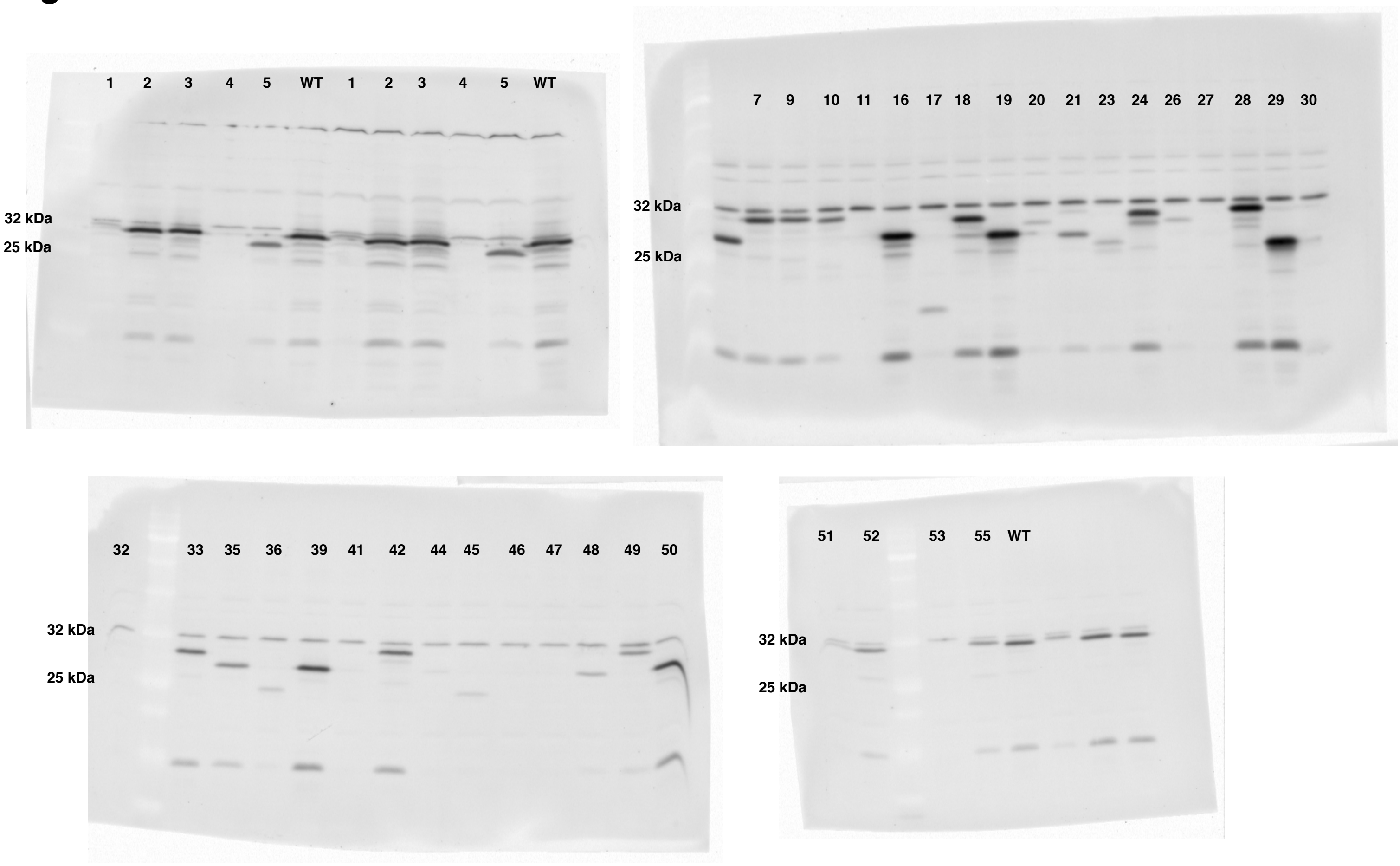

Figure S2. Full immunoblots of Irc6 protein expression in irc6 mutants corresponding to Fig. 1b.

Figure S3

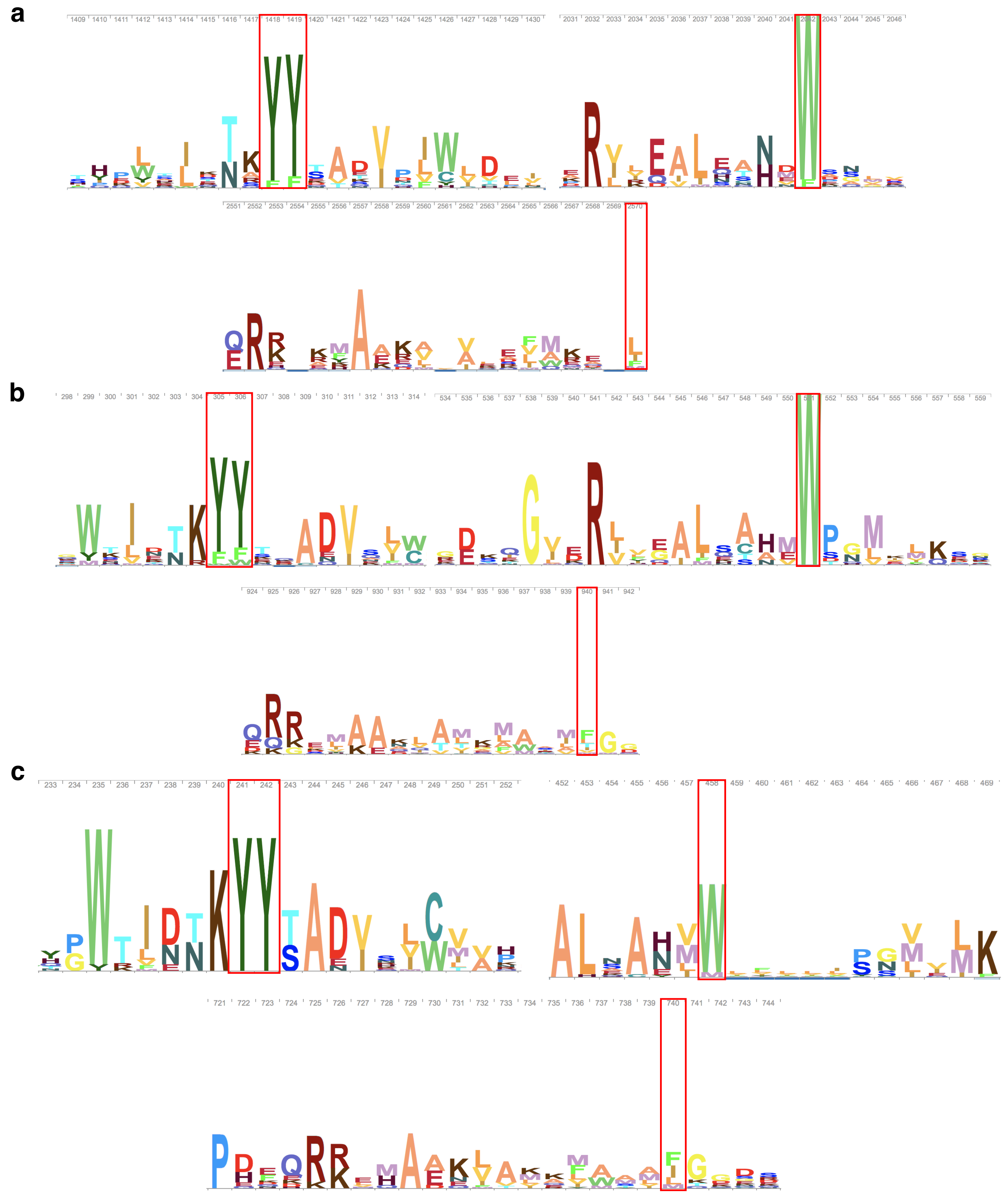

Figure S3. HMM logo representations of the BC-YY motif, conserved tryptophan region, and the C terminal region of the adapin-binding domain from sequence sets retrieved from NCBI protein database using NCBI conserved domain architecture retrieval tool. a) adaptin-binding domain alone (1145 sequences); b) P-loop NTPase and adaptin-binding (167 sequences); c) Ras and adaptin-binding (161 sequences). The red boxes highlight the YY motif, conserved tryptophan, and conserved C-terminal large hydrophobic residue.

Figure S4

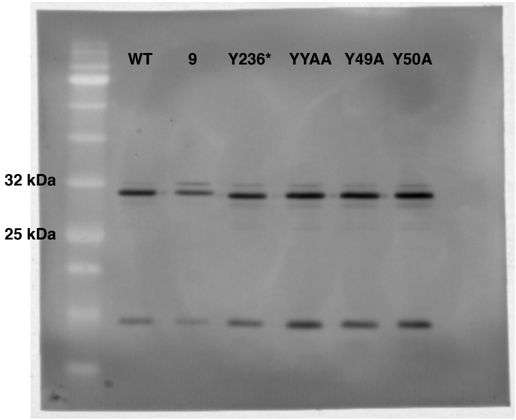

Figure S4. Full immunoblot of Irc6 protein expression in irc6 mutants corresponding to Fig. 2c.

Figure S5

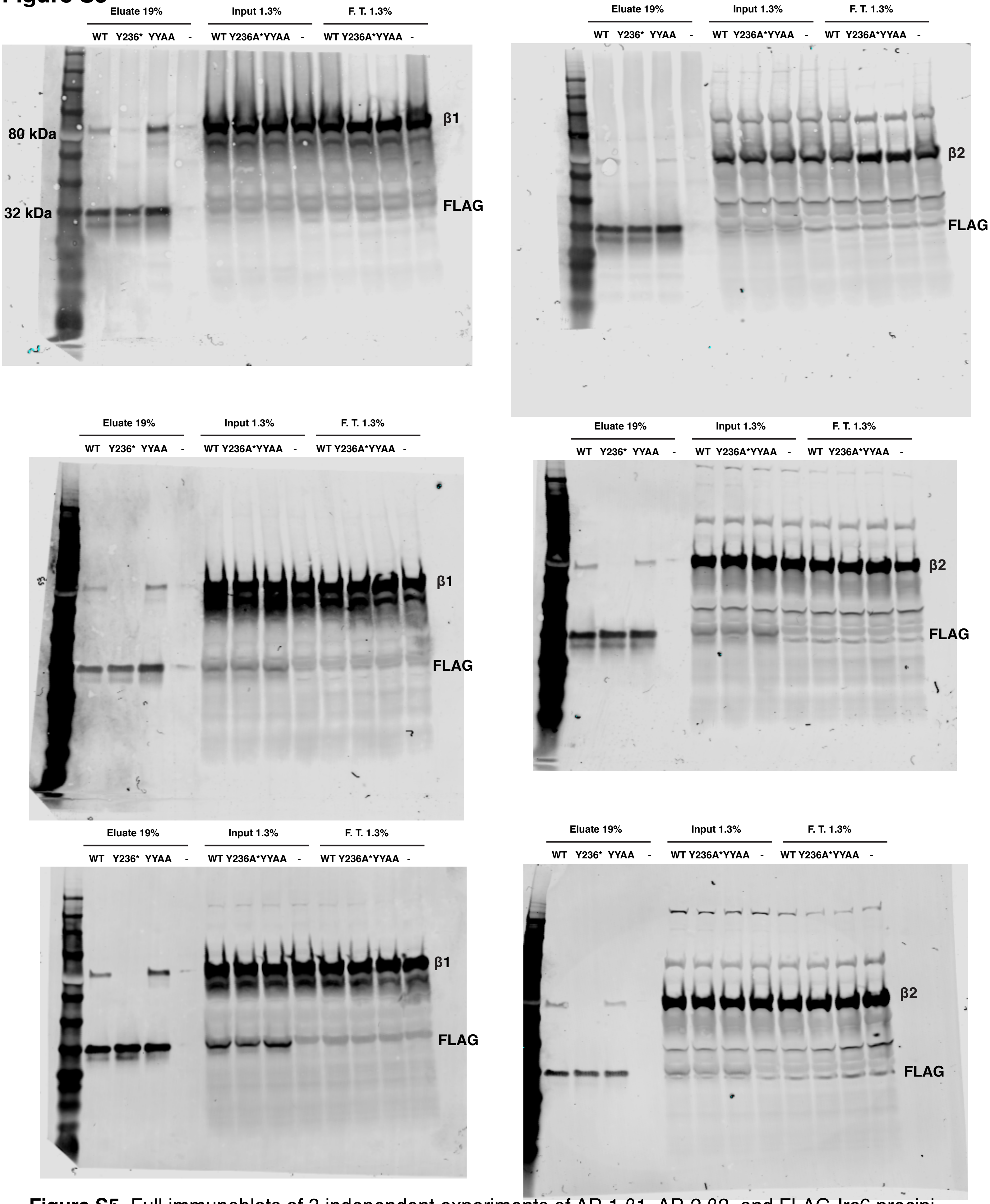

Figure S5. Full immunoblots of 3 independent experiments of AP-1 β1, AP-2 β2, and FLAG-Irc6 precipitated with FLAG antibody, corresponding to Fig. 3d. Eluate represents immunoprecipitated proteins, Input represents the total lysate, F.T. (flow-through) represents extract proteins remaining after immunoprecipitation. Amounts analyzed in each lane as percentages of the original lysate volume are shown.

Figure S6

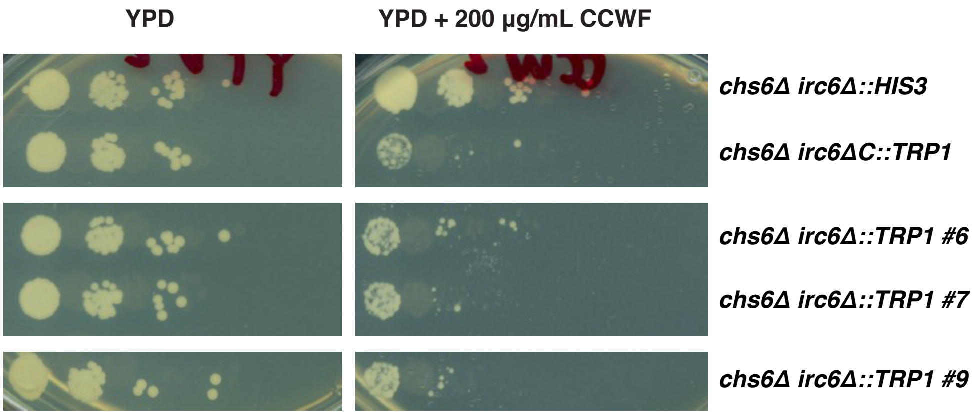

Figure S6. Selectable marker effect on CCFW sensitivity. The indicated strains were analyzed for CCFW sensitivity as in Fig. 4; *chs6Δ irc6Δ::HIS3* (GPY4042), *chs6Δ irc6ΔC::TRP1* (GPY4993), *chs6Δ irc6ΔC::TRP1 #6, #7, #9* (GPY5087-5089).

Figure S7

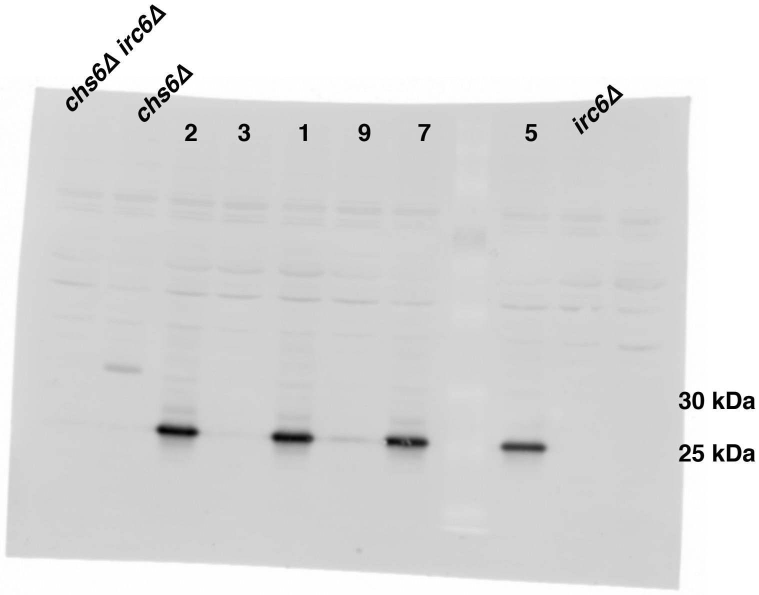

Figure S7. Full immunoblot of Irc6 in yeast extracts from *IRC6* (*chs6Δ*, GPY3102), *chs6Δ GPDp-irc6ΔC* strains (#1,2,3,5,7,9), *chs6Δ irc6Δ* (GPY4042), and *irc6Δ* (GPY4986), corresponding to Fig. 4d.

**Table S1**. Strains used in this study.		
**Strain number**	**Genotype**	**Reference**
GPY404.2	MATa ura3-52, leu2-3,112 his3-Δ200 trp1-Δ901 lys2-801 suc2-Δ9	31
GPY3102	MATa ura3-52, leu2-3,112 his3-Δ200 trp1-Δ901 lys2-801 suc2-Δ9 chs6Δ::HIS3	1
GPY3103	MATalpha ura3-52, leu2-3,112 his3-Δ200 trp1-Δ901 lys2-801 suc2-Δ9 chs6Δ::HIS3 apl2Δ::TRP1	1
GPY3109	MATalpha ura3-52 leu2-3,112 his3-Δ200 trp1-Δ901, lys2-801 suc2-Δ9 APL2-GFP::His3MX6 GGA2-mRFP::KanMX	14
GPY4056	MATalpha ura3-52 leu2-3 -112 his3-Δ200 trp1-Δ190, lys2-801 suc2-Δ9 irc6Δ::HIS3MX6 APL2-GFP::HIS3MX6 GGA2-RFP::KANMX6	this study
GPY4993	MATalpha ura3-52 leu2-3,112 his3-Δ200 trp1-Δ901 lys2-801 suc2-Δ9 chs6Δ::HIS3 irc6ΔC::TRP1	1
GPY4042	MATalpha ura3-52 leu2-3 -112 his3-Δ200 trp1-Δ901, lys2-801, suc2-Δ9 irc6Δ::HIS3MX6 chs6Δ::HIS3MX6	1
GPY5086	MATalpha ura3-52 leu2-3,112 his3-Δ200 trp1-Δ901 lys2-801 suc2-Δ9 chs6Δ::HIS3 irc6Δ::TRP1	this study
GPY5087	MATalpha ura3-52 leu2-3,112 his3-Δ200 trp1-Δ901 lys2-801 suc2-Δ9 chs6Δ::HIS3 irc6Δ::TRP1	this study
GPY5088	MATalpha ura3-52 leu2-3,112 his3-Δ200 trp1-Δ901 lys2-801 suc2-Δ9 chs6Δ::HIS3 irc6Δ::TRP1	this study
GPY5089	MATalpha ura3-52 leu2-3,112 his3-Δ200 trp1-Δ901 lys2-801 suc2-Δ9 chs6Δ::HIS3 irc6Δ::TRP1	this study
GPY4986	MATa ura3-52 leu2-3,112 his3-Δ200 trp1-Δ901 lys2-801 suc2-Δ9 irc6Δ::URA3	1
GPY5090	MATalpha ura3-52 leu2-3,112 his3-Δ200 trp1-Δ901 lys2-801 suc2-Δ9 chs6Δ::HIS3 irc6ΔC	this study
GPY5091	MATa ura3-52 leu2-3,112 his3-Δ200 trp1-Δ901 lys2-801 suc2-Δ9 chs6Δ::HIS3 irc6ΔC	this study
GPY5092	MATalpha ura3-52 leu2-3,112 his3-Δ200 trp1-Δ901 lys2-801 suc2-Δ9 chs6Δ::HIS3 irc6ΔC	this study
GPY5093	MATalpha ura3-52 leu2-3,112 his3-Δ200 trp1-Δ901 lys2-801 suc2-Δ9 chs6Δ::HIS3 Kan-GPDp-irc6ΔC::TRP1	this study
GPY5094	MATalpha ura3-52 leu2-3,112 his3-Δ200 trp1-Δ901 lys2-801 suc2-Δ9 chs6Δ::HIS3 Kan-GPDp-irc6ΔC::TRP1	this study

Table S2. Primers used in this study

Primer #	Primer Name	Sequence (5' to 3')	Length	Description
HZ15	Icr6-F	ATGGTTCTTCAATACCCCCAGAAT	24	to construct irc6ΔC without marker
HZ16	Irc6-179-R	TTATTGGTTTAATACATATGTATACACATATACATATCTGTACA TACTCAtaaccagtcgtgggtatctataatc	75	to construct irc6ΔC without marker. Lower case indicates sequences homologous to *IRC6* to generate truncation at aa179.
HZ17	Irc6-S1	AATCTGATGCAGCAAGATAGCAAGTATATATACGCAAAAATA CCAATCTACCATGcgtacgctgcaggtcgac	73	to construct GPDp-irc6ΔC. Lower-case sequence is the S1 primer sequence in ref. 28.
HZ18	Irc6-S4	AGTTGTGTGGGTGATCTGATAAAACCAATATTTTATTCTGGGG GTATTGAAGAACcatcgatgaattctctgtcg	75	to construct GPDp-irc6ΔC. Lower-case sequence is the S4 primer sequence in ref. 28.
HZ87	Irc6delF1 new	CAAGATAGCA AGTATATATA CGCAAAAATA CCAATCTACC cggatccccgggttaattaa	64	to construct irc6Δ with TRP1 marker. Red font indicates the F1 primer sequence in ref. 29.
HZ88	Irc6delR1 new	TTTAATACATATGTATACACATATACATATCTGTACATAC gaattcgagctcgtttaaac	62	to construct irc6Δ with TRP1 marker. Red font indicates the R1 primer sequence in ref. 29.
HZ136	Irc6 Up F SpeI	ATAA actagt AAGTCCTTGAACTCTCCAAACA	34	500bp upstream of Irc6 start codon SEY6210. Red font is SpeI site.
HZ137	Irc6 Down R XmaI	ATAA cccggg ATGTTTTGTTCCAGCATCATGG	34	500bp downstream of Irc6 stop condon. Red font is XmaI site.
HZ138	3xFLAG-Irc6 F	GATTATAAAGATCATGACATCGATTACAAGGATGACGATGAC AAG GGTGGT ATGGTTCTTCAATACCCCCAG	74	to insert 3xFLAG at the Irc6 N terminus. The 3xFLAG sequence is MDYKDHDGDYKDHDIDYKDDD DKGGM… Two glycines and methionine are included as a linker. Red font indicates Irc6 sequence. This primer anneals with reverse primer to generate the full 3xFLAG sequence. Green font indicates sequences that anneal to reverse primer. Blue font indicates codons for two glycines between FLAG tag and start codon.
HZ139	3xFLAG-Irc6 R	GTAATCGATGTCATGATCTTTATAATCACCGTCATGGTCTTTGT AGTCCAT TGTAGATTGGTATTCTTGCGTATAT	77	to insert 3xFLAG at the Irc6 N terminus. The 3xFLAG sequence is MDYKDHDGDYKDHDIDYKDDD DKGGM… Red font indicates sequences homologous to *IRC6* promoter region. This primer anneals with forward primer to generate the full 3xFLAG sequence. Green font indicates sequences that anneal to forward primer
HZ140	Irc6 435bp F	CGTAAACTGGAAAAGGTCAAAAC	23	for random mutagenesis at Irc6 C term, starting at Lys152
HZ141	Irc6 814bp R	ACTTCCCTGTACTACTAACAGC	22	for random mutagenesis at Irc6 C term, starting from 100bp downstream of stop codon

**Table S3.** Genes associated with GO terms in negative genetic interaction screens		
Irc6Δ		
**GO ID**	**GO term**	**Genes**
GO:0006888	ER to Golgi vesicle-mediated transport	BOS1,SEC13,SEC22,COP1,CDC1,TRS23,SEC27,YKT6,BET3,BET5,SEC15,CMD1,ARC35, RSP5,BRR6,KAR2,TEM1
GO:0006458	'de novo' protein folding	CCT6,TCP1,KRE5,ROT1,STT3,CDC1,GPI12,SRV2
GO:0051274	beta-glucan biosynthetic process	KRE9,KAR2,UGP1,KRE5,PKC1,ROT1,LST8
GO:0098781	ncRNA transcription	RPC11,RRN7,RPC17,RPC34,MOT1
GO:0006898	receptor-mediated endocytosis	CMD1,ARC35,RSP5,CDC24,PKC1,TEM1,ROT1,SRV2,LST8,BOS1,SEC22,BRN1,YKT6, SPT16,MOT1,SEC13
GO:0006281	DNA repair	SSL1,NSE1,POL3,CDC1,TFB1,SPT16,ARP4,POL31,ARP7,BRN1,ORC2,SEC13,KAR2,KRE5
GO:0006351	transcription, DNA-templated	SSL1,SEC13,KIN28,RPC11,TFB1,ORC2,SPT16,PTI1,ARP4,RRN7,RPC17,RPC34,MOT1, ARP7,RSP5,PKC1
GO:0006379	mRNA cleavage	RPC11,PTI1,HRP1,KIN28
GO:0070816	phosphorylation of RNA polymerase II C-terminal domain	SSL1,KIN28,TFB1,RRN7,MOT1
GO:0051345	positive regulation of hydrolase activity	SEC13,CDC24,TRS23,BRN1,BET3,BET5,LST8,PKC1,CMD1,RSP5,SRV2
GO:0051301	cell division	CDC24,KIN28,CDC1,PKC1,BRN1,CMD1,TEM1,ROT1
GO:0006890	retrograde vesicle-mediated transport, Golgi to ER	SEC22,COP1,SEC27
GO:0006997	nucleus organization	SEC13,CMD1,BRR6,KAR2
GO:0006661	phosphatidylinositol biosynthetic process	CDC1,CMD1,GPI12
GO:0018193	peptidyl-amino acid modification	NSE1,PKC1,ORC2,STT3,ARP4,KRE5
apm1Δ		
**GO ID**	**GO term**	**Genes**
GO:0016192	vesicle-mediated transport	STP22,ACT1,YPT1,VPS54,CDC1,SEC26,TRS23,SWA2,TLG1,SLA1,TRS20,SEC15,BET4, VPS53,TRS33,LAS17,MRS6,PAN1,SEC2,SLA2,TPM1,SEC23,UBP3,SEH1,HSP10,NUP159, CSL4,TIF6,HUR1
GO:0006596	polyamine biosynthetic process	SPE1,SPE2,SPE3,CHO2,ZWF1,FMN1
GO:0000147	actin cortical patch assembly	SLA1,LAS17,PAN1,SLA2,SRV2,TPM1,ACT1,SPC42,SPC97,MGM1,SWA2,MDM20,CDC12, TLG1
GO:0043933	protein-containing complex subunit organization	YPT1,SMD2,SWA2,SLA1,SWC5,VMA21,SPC42,HSP10,NOB1,LAS17,MGM1,PAN1,CUS1, SLA2,SRV2,DCP2,NOP2,TIF6,SEC23,CDC12,SPC97,SOM1,MCM3
GO:0046467	membrane lipid biosynthetic process	PER1,LCB2,CDC1,LIP1,GPI2,SUR1,ERG3,CHO2,BTS1
GO:0006887	exocytosis	ACT1,SEC15,SEC2,SLA2,TPM1,YPT1,TLG1,MDM20,NUP159,TIF6,SEC23
GO:0042147	retrograde transport, endosome to Golgi	YPT1,VPS54,TLG1,VPS53
GO:0006396	RNA processing	RPL8B,CFT2,SMD2,TAD3,FAL1,RRP8,UTP5,PTI1,NOP19,NOB1,GEP3,CUS1,CSL4,DCP2, NOP2,TIF6,SNT309,UTP9
